# The Metabolic Heart: Reframing Heart Failure With Preserved Ejection Fraction as a Systemic Cardio-Metabolic Syndrome

**DOI:** 10.7759/cureus.100590

**Published:** 2026-01-01

**Authors:** Debasrita Baidya, Abhishek Hanumanpratap Singh Kshatri, Emi K Zerzan, Gayathri J Menon, Mihika Sawale, Sunny Subhash Bhalodiya

**Affiliations:** 1 Biomedical Sciences, Indian Institute of Technology Patna, Bihta, IND; 2 Emergency Medicine, Apollo Hospitals Health City, Visakhapatnam, IND; 3 College of Natural Science, The University of Texas at Austin, Texas, USA; 4 Cardiology, Dr. Chandramma Dayanand Sagar Institute of Medical Education and Research (CDSIMER), Bengaluru South District, IND; 5 Internal Medicine, K.J. Somaiya Medical College and Research Centre, Mumbai, IND; 6 Internal Medicine, Hinduhrudaysamrat Balasaheb Thackarey Medical College, Mumbai, IND

**Keywords:** cardio-metabolic syndrome, epicardial adipose tissue, glp-1 receptor agonists, heart failure with preserved ejection fraction, insulin resistance, metabolic heart disease, mitochondrial dysfunction, precision medicine, sglt2 inhibitors, systemic inflammation

## Abstract

This review critically reassesses heart failure with preserved ejection fraction (HFpEF) as a systemic cardio-metabolic disorder. Searching PubMed, Scopus, and Web of Science (2000-2025) yielded 108 priority studies, randomized controlled trials (RCTs), prospective cohorts, and mechanistic reports after screening ~800 records. Evidence shows visceral adiposity, insulin resistance, chronic inflammation and mitochondrial dysfunction synergistically impair cardiac energetics. Unlike conventional neuro-hormonal blockade (renin angiotensin aldosterone system or RAAS inhibitors, beta-blockers), which showed neutral outcomes in heterogeneous HFpEF populations, landmark RCTs (EMPagliflozin outcomE tRial in Patients With chrOnic heaRt Failure With Preserved Ejection Fraction (EMPEROR-Preserved), Dapagliflozin Evaluation to Improve the Lives of Patients With Preserved Ejection Fraction Heart Failure (DELIVER)) now demonstrate that sodium-glucose cotransporter-2 inhibitors cut hospitalizations and cardiovascular events, while glucagon-like peptide-1 (GLP-1) receptor agonists improve symptoms, exercise tolerance and weight, validating the metabolic-inflammation paradigm. Persistent phenotypic heterogeneity underscores the need for biomarker-driven phenotyping, advanced imaging and adaptive trial designs to embed metabolic and bioenergetic dimensions into early, precision care.

## Introduction and background

This narrative review was guided by a comprehensive, non-systematic literature search of PubMed, Scopus, and Web of Science (2000-2025). Discrete keyword sets were constructed for each thematic section (mechanistic, diagnostic, and therapeutic) to capture the breadth of contemporary guidelines, translational studies, and clinical trials; no unified search string or formal screening algorithm was applied. Heart failure (HF) is a clinical syndrome in which abnormal cardiac function leads to signs and symptoms such as dyspnea and fatigue due to impaired filling and ejection of blood [1,2]. Classification is based on the left ventricular ejection fraction (LVEF), which influences prognosis and treatment response [1,2]. The 2022 American Heart Association, American College of Cardiology, Heart Failure Society of America (AHA/ACC/HFSA), 2017 Canadian Cardiovascular Society (CCS), and 2021 European Society of Cardiology (ESC) guidelines define HF with preserved ejection fraction (HFpEF) as LVEF ≥50% [1-3]. Although both AHA/ACC/HFSA and ESC define HFpEF as ejection fraction (EF) ≥50%, the ESC retains a distinct HF with Mildly Reduced Ejection Fraction (HFmrEF) category (EF 41-49%), whereas CCS requires objective evidence of elevated filling pressures during exercise or volume challenge when resting hemodynamics are equivocal, and National Institute for Health and Care Excellence (NICE) mandates at least one structural (left atrial enlargement or left ventricular hypertrophy (LVH)) plus one functional echocardiographic criterion (E/e′ ≥15 or average e′ <7 cm s⁻¹) [1,3,4].

HFpEF as a cardiovascular disease

According to the National Health and Nutrition Examination Survey (NHANES) 2017-2020, approximately 6.7 million U.S. adults (≥20 years) have HF. HFpEF accounts for more than half of these cases and is associated with substantial morbidity and mortality [5]. The lifetime risk of HFpEF was estimated to be 9.3% from 1990 to 2014 [6]. The five-year mortality rate for HFpEF was 75.7% in the Get With The Guidelines (GWTG) Registry, which included 39,982 hospitalized HF patients with Medicare follow-up from 2005 to 2014 [6,7]. Clinically, HFpEF presents with breathlessness, dyspnea, reduced exercise tolerance, fatigue, and swelling, although symptoms alone cannot differentiate it from other types of heart failure. The frequent coexistence of comorbidities further complicates the attribution of symptoms to HFpEF [5]. Identifying specific etiologies, such as cardiac amyloidosis, hypertrophic cardiomyopathy, sarcoidosis, hemochromatosis, or Fabry disease, supports targeted treatment [5].

The 2022 AHA/ACC/HFSA guidelines require elevated left ventricular filling pressures at rest, during exercise, or during provocation for HFpEF diagnosis. This can be established by increased natriuretic peptide levels (N-terminal pro-B-type natriuretic peptide (NT-proBNP) >125 pg/mL, B-type natriuretic peptide (BNP) ≥35 pg/mL), echocardiography, or invasive hemodynamic measurements. Structural alterations, such as left atrial enlargement, can further support this diagnosis [1]. Despite these criteria, HFpEF is frequently under-recognized, particularly in older adults [8]. The limited use of diagnostic tools such as natriuretic peptide testing and echocardiography has also led to overdiagnosis in some cases [9]. 

The current management strategies recommended by the 2022 AHA/ACC/HFSA Guidelines include general HF therapy with diuretics [2], etiology-specific treatments, and comorbidity management [1]. Sodium-glucose cotransporter-2 (SGLT2) inhibitors have a Class of Recommendation 2a (COR 2a). The EMPagliflozin outcomE tRial in Patients With chrOnic heaRt Failure With Preserved Ejection Fraction (EMPEROR-Preserved) trial demonstrated that empagliflozin reduced the composite of cardiovascular death or hospitalization [10], whereas the Pharmacotherapies in Heart Failure with PRESERVED Ejection Fraction in Heart Failure (PRESERVED-HF) trial showed that dapagliflozin improved symptoms, physical function, and exercise capacity [11]. Angiotensin Receptor Blockers (ARBs), Angiotensin Receptor-Neprilysin Inhibitors (ARNIs), and Mineralocorticoid Receptor Antagonists (MRAs) carry a Class of Recommendation 2b (COR 2b), reflecting weaker evidence [1]. In Prospective Comparison of ARNI with ARB Global Outcomes in Heart Failure with Preserved Ejection Fraction (PARAGON-HF), sacubitril/valsartan did not significantly lower the rates of hospitalization or cardiovascular death [12], while in Treatment of Preserved Cardiac Function Heart Failure With an Aldosterone Antagonist Trial (TOPCAT), spironolactone did not significantly reduce the combined outcome of cardiovascular death, cardiac arrest, and hospitalization [13]. The heterogeneity of HFpEF across pathophysiology, filling pressures, and comorbidities likely underlies these trial failures, underscoring the need for phenotype-based approaches [14]. These definitional discrepancies influence trial enrollment and external validity: EMPEROR-Preserved permitted inclusion down to 40% EF under ESC criteria, whereas AHA 2022 sites reclassified the same range as HFmrEF, contributing to divergent baseline phenotypes across trials [10]. HF with LVEF ≤40% is termed heart failure with reduced ejection fraction (HFrEF), and 40% < LVEF < 50% is classified as HFmrEF [1-3]. The NICE guidelines further explain that HFpEF is usually associated with impaired ventricular relaxation rather than contraction [4]. 

 HFpEF as a whole-body disease

The 2022 AHA/ACC/HFSA guidelines emphasize the strong association between HFpEF and comorbidities, such as obesity, hypertension, diabetes, coronary artery disease, and chronic kidney disease [1]. More than 80% of patients with HFpEF are overweight or obese. The Study of the Effect of Calorie Restriction and Exercise Training (SECRET) trial demonstrated that caloric restriction or aerobic training elevated peak V̇O₂ in obese individuals [15]. Diabetes further worsens HFpEF prognosis, as shown in the Phosphodiesterase-5 Inhibition to Improve Clinical Status and Exercise Capacity in Heart Failure with Preserved Ejection Fraction (RELAX) trial, where patients with diabetic HFpEF experienced a higher hospitalization risk and reduced exercise capacity due to multimorbidity, impaired chronotropic reserve, LVH, and inflammation [16]. A meta-analysis by Halabi et al. (2020) demonstrated a reduction in mortality with metformin in patients with HFpEF [17]. 

While earlier research emphasized cardiac dysfunction and conventional risk factors such as hypertension, recent studies have highlighted metabolic abnormalities, oxidative stress, inflammation, and mitochondrial dysfunction as the central drivers of HFpEF. Thus, HFpEF management requires comprehensive systemic approaches, with metabolic regulation and comorbidity control as the cornerstones of therapy [1,18]. Recognizing HFpEF as a cardio-metabolic syndrome not only provides a descriptive advantage, but it also immediately justifies multi-organ phenotype-based trials, explains why purely cardiac-directed therapies have underperformed, and positions SGLT2 inhibitors and glucagon-like peptide (GLP)-1 receptor agonists, agents originally developed for metabolic disease, as disease-modifying therapies for HFpEF.

## Review

How metabolism affects the heart in HFpEF

HFpEF accounts for nearly half of all HF cases worldwide, affecting approximately 64 million individuals. Its prevalence is projected to increase by approximately 46% by 2030 owing to population aging and rising metabolic diseases [19]. Unlike HFrEF, HFpEF is characterized by preserved left ventricular systolic function but presents with exertional dyspnea, fatigue, and exercise intolerance. It occurs more commonly in older adults, women, and individuals with metabolic comorbidities, such as obesity, type 2 diabetes mellitus (T2DM), and hypertension [20]. 

A central concept in HFpEF pathogenesis is the “outside-in” paradigm, in which metabolic and inflammatory disturbances originating in extracardiac tissues, including adipose tissue, liver, skeletal muscle, and vasculature, promote systemic inflammation, endothelial dysfunction, and insulin resistance. These processes impair myocardial and vascular homeostasis, leading to clinical manifestations of HFpEF [21]. In obesity and T2DM, dysfunctional adipose tissue secretes pro-inflammatory mediators (tumor necrosis factor-alpha (TNF-α), interleukin-6 (IL-6), and monocyte chemoattractant protein-1 (MCP-1)), driving endothelial activation of vascular cell adhesion molecule-1 (↑VCAM-1) & endothelial nitric oxide synthase (↓eNOS), coronary microvascular inflammation, rarefaction, myocardial fibrosis, and diastolic stiffness, which are hallmarks of HFpEF [22]. The EMPEROR-Preserved trial (2021; n=5,988; LVEF >40%) showed that empagliflozin reduced the risk of cardiovascular death or HR hospitalization by 21% (HR 0.79, 95% CI 0.69-0.90, p<0.001) [10]. This shows how important metabolic dysregulation is in HFpEF.

Similarly, the Dapagliflozin Evaluation to Improve the LIVEs of Patients With PReserved Ejection Fraction Heart Failure or DELIVER trial (2022; n=6,263; LVEF >40%) demonstrated an 18% reduction in the same endpoint with dapagliflozin (HR 0.82, 95% CI 0.73-0.92, p<0.001) [23]. These results are significant because they show that targeting cardiorenal-metabolic pathways through glycosuria, natriuresis, improved myocardial energetics, and anti-inflammatory effects changes the course of HFpEF disease, where neurohormonal blockade had previously failed. Importantly, the benefits were consistent irrespective of diabetes status, highlighting that the metabolic foundation of HFpEF extends beyond hyperglycemia to insulin resistance, abnormal substrate utilization, and chronic inflammation [24]. Given that up to 80% of patients with HFpEF have metabolic comorbidities [21], metabolic derangements must be considered as the central causal mechanisms. SGLT2 inhibition has emerged as the first proven disease-modifying approach for HFpEF, thus validating the outside-in paradigm [21,24]. 

Myocardial metabolic remodeling

In healthy states, the heart exhibits metabolic flexibility, adapting fuel use (fatty acids vs. glucose) to meet adenosine triphosphate (ATP) demand under varying physiological conditions [25]. Approximately 60-70% of cardiac ATP is derived from the mitochondrial β-oxidation of long-chain fatty acids, whereas 30-40% originates from glucose and lactate, allowing efficient contractile support and oxygen economy [26]. However, this adaptability is lost in patients with HFpEF. The myocardium shifts toward increased fatty acid oxidation, reduced glucose uptake (due to impaired glucose transporter type 4 (GLUT4) translocation), and diminished mitochondrial oxidative capacity [25,27]. This maladaptive remodeling results in lipotoxicity (ceramides and diacylglycerols), oxidative stress, mitochondrial dysfunction, and impaired cardiomyocyte relaxation, ultimately leading to diastolic stiffness [27,28].

Biopsy studies by van Heerebeek et al. (2012) revealed reduced myocardial protein kinase G (PKG) activity and titin hypophosphorylation, which increased myocardial stiffness [29]. Schiattarella et al. (2019) identified nitrosative stress and impaired unfolded protein response (UPR) signaling, linking metabolic inflammation to fibrosis [30]. Scandalis et al. (2023) recently showed a strong link between mitochondrial and energetic deficits, exercise intolerance, and the severity of symptoms in HFpEF [31].

Adipose tissue inflammation and insulin resistance

Most patients with HFpEF present with central obesity, metabolic syndrome, and/or T2DM [19,32]. Adipose tissue in these states is enriched with pro-inflammatory macrophages that release cytokines such as TNF-α, IL-6, and MCP-1, which drive systemic inflammation, endothelial dysfunction, and insulin resistance [33]. Epicardial adipose tissue (EAT) exhibits increased pro-inflammatory activity. Zhao et al. (2020) and Cho and Park (2024) reported 1.8 to 2.5-fold higher IL-6 and TNF-α expression in EAT compared with subcutaneous fat in patients undergoing cardiac surgery [34,35]. Macrophages from insulin-resistant adipose tissue produce significantly more pro-inflammatory cytokines than those from lean tissue, directly linking adipose inflammation to metabolic and cardiovascular diseases [36,37]. Li et al. (2020) further confirmed that adipose inflammation correlates with impaired endothelial-dependent vasodilation in obese patients with HFpEF [38].

Insulin resistance exacerbates metabolic remodeling by reducing myocardial glucose uptake and increasing fatty acid oxidation and lipotoxicity [39]. This cascade amplifies oxidative stress, disrupts sarcomere relaxation, and advances diastolic dysfunction [40].

EAT and cardiac dysfunction

The EAT directly influences the heart via paracrine and mechanical mechanisms, as it is located between the myocardium and visceral pericardium without a fascial barrier [35,41]. Li et al. (2024) showed that a 2-3 mm increase in EAT thickness correlates with impaired diastolic function, even in normotensive individuals [42]. The PROMIS-HFpEF cohort demonstrated that greater EAT thickness (≥9 mm) was independently associated with impaired coronary flow reserve, with a 33% higher odds of impairment per standard deviation increase in EAT volume (OR, 1.33; 95% CI, 1.02-1.73; p=0.03) [43]. Similarly, Maimaituxun et al. (2021) reported that a higher EAT volume was associated with reduced global longitudinal strain (− 15.3% vs. − 17.1%; p<0.001), indicating subclinical systolic dysfunction despite preserved EF [30,37,44].

Systemic vascular dysfunction and endothelial inflammation

Systemic inflammation and metabolic stress impair microvascular endothelial function, which is central to the pathogenesis [30,37]. Simmonds et al. (2020) demonstrated reduced eNOS expression, macrophage infiltration, and perivascular fibrosis in myocardial biopsies of patients with HFpEF [45]. In PRevalence Of MIcrovascular dySfunction in Heart Failure with Preserved Ejection Fraction (PROMIS-HFpEF), nearly 75% of patients had a reduced coronary flow reserve (<2.5), which predicts adverse outcomes [43]. Radakrishnan et al. (2025) demonstrated that myocardial perfusion abnormalities in women with HFpEF were associated with pathological left ventricular remodeling and exercise intolerance, especially in individuals with visceral adiposity [46]. HFpEF is recognized as a systemic metabolic-inflammatory syndrome defined by the synergistic effects of myocardial remodeling, adipose inflammation, EAT expansion, and vascular dysfunction [23,30]. New treatments, such as SGLT2 inhibitors, GLP-1 receptor agonists, and anti-inflammatory drugs, may change these pathways [46]. Nonetheless, impaired substrate utilization and mitochondrial dysfunction highlight that the heart with HFpEF is energetically starved, linking systemic stress to cardiac energy depletion.

**Figure 1:**
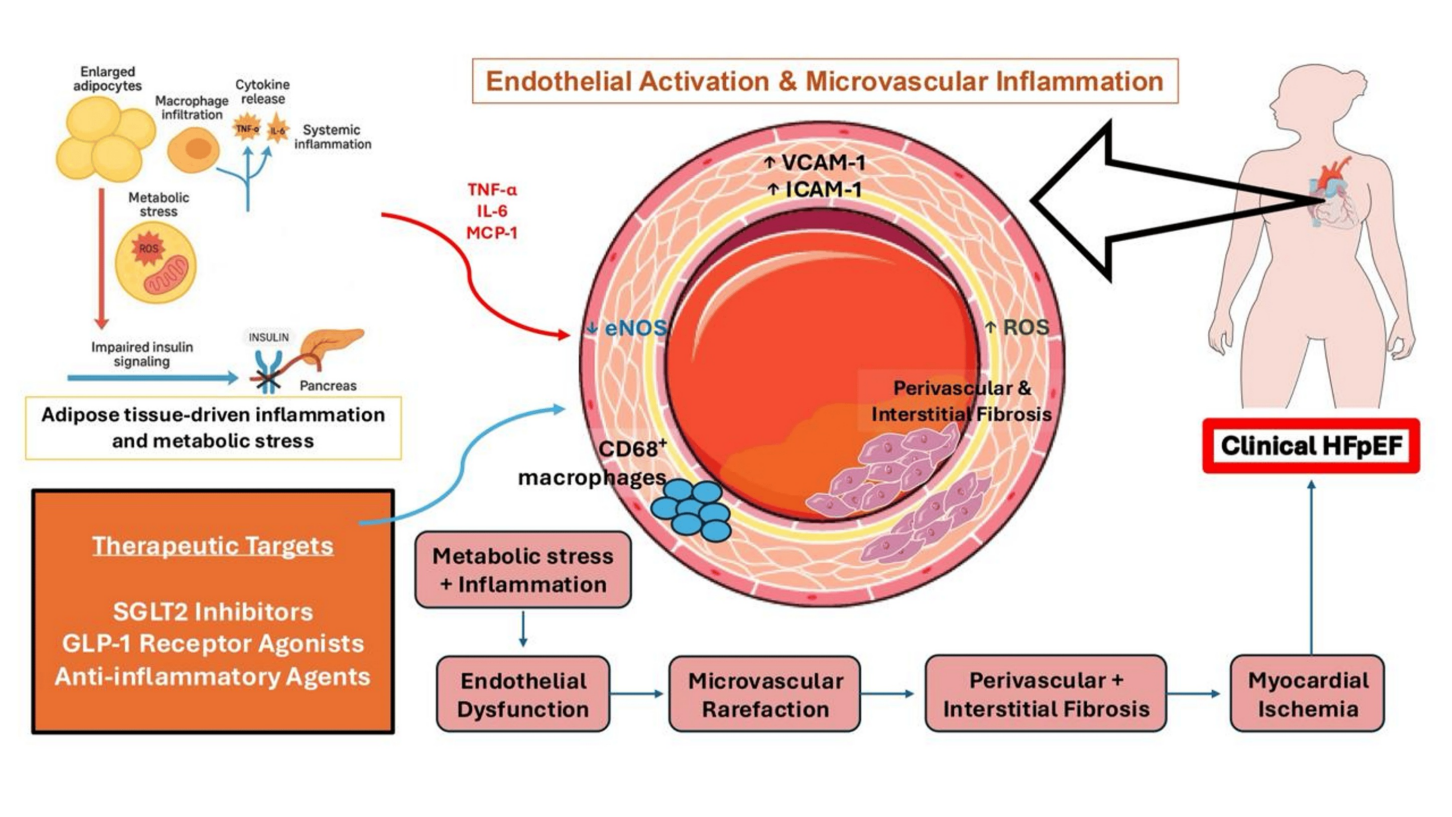
Adipose inflammation–induced endothelial activation drives microvascular dysfunction in HFpEF Inflamed adipose tissue secretes TNF-α, IL-6, and MCP-1, promoting endothelial activation characterized by ↓eNOS, ↑ROS, and adhesion molecule expression. CD68⁺ macrophage infiltration leads to perivascular and interstitial fibrosis, microvascular rarefaction, and ischemia, contributing to HFpEF. Therapeutic targets modulate upstream metabolic and inflammatory triggers [31]. HFpEF: Heart Failure with Preserved Ejection Fraction; TNF-α: Tumor Necrosis Factor-alpha; IL-6: Interleukin-6; MCP-1: Monocyte Chemoattractant Protein-1; ROS: Reactive Oxygen Species; eNOS: Endothelial Nitric Oxide Synthase; VCAM-1: Vascular Cell Adhesion Molecule-1; ICAM-1: Intercellular Adhesion Molecule-1; CD68⁺ Macrophages: Cluster of Differentiation 68-positive Macrophages; SGLT2 Inhibitors: Sodium-Glucose Cotransporter-2 Inhibitors; GLP-1 RAs: Glucagon-Like Peptide-1 Receptor Agonists Image credit: Adapted from image provided by Servier Medical Art (https://smart.servier.com), licensed under CC BY 4.0 (https://creativecommons.org/licenses/by/4.0/).

Adipose inflammation-induced endothelial activation drives microvascular dysfunction in HFpEF. Inflammatory adipose tissue releases TNF-α, IL-6, and MCP-1, resulting in decreased eNOS, increased ROS, elevated adhesion molecule expression, macrophage infiltration, fibrosis, microvascular rarefaction, and ischemia (Figure 1) [31].

Table 2 summarizes key studies investigating inflammation, metabolism, and EAT in HFpEF pathogenesis.

**Table 1:** Key studies investigating inflammation, metabolism, and EAT in HFpEF pathogenesis FA: Fatty Acid;  ATP: Adenosine Triphosphate; HFpEF: Heart Failure with Preserved Ejection Fraction; HFrEF: Heart Failure with Reduced Ejection Fraction; IR: Insulin Resistance PKG: Protein Kinase G; RHI: Reactive Hyperemia Index; EAT: Epicardial Adipose Tissue; UPR: Unfolded Protein Response; IL-6: Interleukin-6; TNF-α: Tumor Necrosis Factor-alpha;  CABG: Coronary Artery Bypass Grafting; LVEDP: Left Ventricular End-Diastolic Pressure; Echo: Echocardiography; MRI: Magnetic Resonance Imaging.

Study (Year)	Design	Population/Model	Key Findings	Clinical Relevance
Lopaschuk et al. (2010) [25]	Review/experimental	Cardiac tissue, models	FA β-oxidation is main ATP source in health	Shift in substrate use in HFpEF
Spencer et al. (2010) [36]	Adipose tissue profiling	Obese/insulin-resistant	Macrophage ↑cytokines in IR vs lean tissue	Inflammatory adipose tissue
Van Heerebeek et al. (2012) [29]	Myocardial biopsy	HFpEF vs HFrEF patients	↓PKG, titin hypophosphorylation, ↑stiffness	Energetic remodeling in HFpEF
Brestoff and Artis (2015) [37]	Clinical study	Obese HFpEF patients	↑Inflammation, ↓vasodilation (RHI)	Endothelial dysfunction in HFpEF
Shah et al. (2018) [43]	Echo-observational	HFpEF, preserved EF	↑EAT volume ↔ ↓global longitudinal strain	Subclinical systolic dysfunction
Schiattarella et al. (2019) [30]	Molecular pathway analysis	Human HFpEF myocardium	↓UPR, ↑nitrosative stress, fibrosis	Metabolic-inflammatory HFpEF phenotype
Zhao et al. (2020) and Cho and Park (2024) [34,35]	Ex vivo fat analysis	CABG patients	↑IL-6, TNF-α in EAT vs subcutaneous fat	EAT as inflammatory depot
Maimaituxun et al. (2021) [44]	Invasive hemodynamics	HFpEF during exercise	↑EAT volume ↔ ↑LVEDP, pericardial restraint	Mechanical impact of EAT
Lozhkin et al. (2022) [40]	Echo-correlation	Hypertensive individuals	EAT thickness ↔ diastolic dysfunction	EAT as subclinical marker
Li et al. (2024) [42]	Cardiac MRI, perfusion	HFpEF patients	↑EAT volume ↔ ↓coronary flow reserve	EAT as perfusion modulator
Cho and Park (2024) [35]	Ex vivo fat analysis	EAT and heart failure	EAT inflammation impacts myocardium	EAT as friend or foe in HF
Radakrishnan et al. (2025) [46]	Imaging/clinical cohort	Women with HFpEF	Perfusion deficit linked to remodeling	Sex-specific microvascular impairment

The heart’s energy system: is the engine failing

In HFpEF, the myocardium’s “inside-out” energy failure parallels systemic metabolic stress. Normally, metabolic flexibility-rapid switching among fatty acids, glucose, ketones, and amino acids-lets mitochondria meet ATP demand via oxidative phosphorylation [47]. In HFpEF, this engine malfunctions: fatty-acid oxidation falls, glucose uptake is blunted, and ATP stores drop while reactive oxygen species (ROS) rise, Ca²⁺ handling falters, and mitochondrial dynamics and biogenesis are disrupted [25,48-50]. Unoxidized fatty acids accumulate as toxic lipids, locking the heart into substrate inflexibility that amplifies insulin resistance and contractile decline [51].

Disruption in the powerhouse: intrinsic mitochondrial dysfunction

The mitochondrial electron transport chain (ETC) is where most ATP is made. It is made up of Complexes I-V that are embedded in the inner mitochondrial membrane. Sequential electron transfer generates an electrochemical gradient harnessed by ATP synthase (Complex V) to produce ATP [52]. HFpEF consistently reduces the activity and efficiency of ETC complexes, particularly I-III, which impairs the myocardial energy capacity [52-54].

Electron leakage from the ETC leads to the production of superoxide, which in turn leads to the production of more ROS [55]. ROS damage mitochondrial DNA, proteins, and lipids, further compromising ETC function and creating a vicious cycle of oxidative stress [56,57]. Concurrently, mitochondrial biogenesis is impaired by the downregulation of transcriptional regulators such as Peroxisome Proliferator-Activated Receptor Gamma Coactivator 1-alpha (PGC-1α), leading to the depletion of healthy mitochondria over time [58,59].

The disruption also affected the structural integrity of the mitochondrial network. Normally, a balance between fusion (interconnection) and fission (fragmentation) sustains mitochondrial function. In HFpEF, excessive fission produces fragmented, less efficient, and often pro-apoptotic mitochondria [60-62]. Additionally, mitophagy, the selective removal of damaged mitochondria, is dysregulated, either insufficiently or excessively, causing the accumulation of dysfunctional organelles or the loss of functional mitochondria [63]. These disruptions severely impair oxidative phosphorylation, resulting in insufficient ATP production and progressive myocardial energy depletion [64].

Consequences of the failing energy system: tired heart, tired body

Intrinsic defects in myocardial energetics manifest clinically in HFpEF as diastolic dysfunction and impaired contractile reserve and exercise intolerance.

Diastolic Dysfunction

ATP is necessary for the relaxation of sarcomeres in the heart. Inadequate ATP impairs Sarcoplasmic/Endoplasmic Reticulum Ca²⁺-ATPase 2a (SERCA2a) activity, slowing calcium reuptake into the sarcoplasmic reticulum, prolonging relaxation, stiffening diastole, and elevating left ventricular filling pressure, which are hallmarks of HFpEF [65,66].

Impaired Contractile Reserve and Exercise Intolerance

Despite a preserved ejection fraction at rest, energy deficits limit cardiac output under stress, producing exertional dyspnea and fatigue [67]. Moreover, skeletal muscle mitochondrial dysfunction, which is common in comorbid conditions, contributes to peripheral fatigue and the systemic “tired body” phenotype [68].

Each case of HFpEF is not the same: can we use metabolic profiles?

HFpEF is increasingly recognized as a heterogeneous syndrome rather than a single disease entity. Traditionally, HF has been classified exclusively by the LVEF via echocardiography. However, this approach fails to capture the complex pathophysiology of comorbidities such as diabetes, obesity, atrial fibrillation, renal dysfunction, and systemic inflammation [69]. Therefore, the historical “one-size-fits-all” treatment paradigm is insufficient, prompting efforts to classify HFpEF using phenotypic and metabolic profiles that reflect the underlying mechanisms and patient-specific risk factors.

A systematic review of 20 studies between 2010 and 2025 identified several HFpEF phenotypes, including metabolic-obese, frail-elderly, atrial fibrillation-dominant, cardiorenal, and pulmonary hypertension/right-heart phenotypes. Each exhibits distinct pathophysiological pathways, risk profiles, and responses to therapy, highlighting the inadequacy of EF-based approaches and the potential of precision medicine to improve individualized care [70]. Clinical and etiological classifications further delineate HFpEF subtypes according to the predominant comorbidities, including typical HFpEF associated with diabetes, metabolic syndrome, hypertension, or chronic kidney disease; coronary artery disease-driven HFpEF; atrial fibrillation-predominant HFpEF; right heart failure-predominant HFpEF; hypertrophic cardiomyopathy-like HFpEF; multivalvular HFpEF; and restrictive cardiomyopathies such as amyloidosis or genetic etiologies [69]. Although these classifications are not mutually exclusive, they provide a clinically useful framework for identifying disease mechanisms and tailoring therapies.

Advanced echocardiographic phenotyping, including assessment of global and regional longitudinal strain by speckle tracking, mitral inflow patterns, tissue Doppler imaging, and tricuspid annular plane systolic excursion, enables finer discrimination of cardiac structural and functional abnormalities [69]. For example, a global longitudinal strain (GLS) of less than 15.8% has been shown to predict hospitalization for HF, cardiovascular death, and aborted cardiac arrest in patients with HFpEF. In an Asian cohort, the mean GLS was 13.50 ± 4.00%, and multivariate analysis demonstrated that impaired GLS, higher age-adjusted Charlson Comorbidity Index, and low body mass index (BMI) were independently associated with shorter survival over a three-year follow-up [71].

Epidemiological studies have further reinforced the interplay between metabolic comorbidities and the incidence of HFpEF. In the Multi-Ethnic Study of Atherosclerosis (MESA; n=6,781), over a median follow-up of 11.2 years, 111 participants developed HFpEF (cumulative incidence, 1.7%). Significant predictors include age, hypertension, diabetes, BMI, LVH, interim myocardial infarction, elevated NT-proBNP, detectable troponin T, and left ventricular mass index, with risk patterns largely consistent across racial and ethnic groups [72].

Obesity is highly prevalent and strongly influences the pathogenesis of HFpEF. A secondary analysis of the Semaglutide Treatment Effect in People with obesity and Heart Failure with Preserved Ejection Fraction (STEP-HFpEF) and Semaglutide Treatment Effect in People With Obesity and HFpEF and Type 2 Diabetes Mellitus (STEP-HFpEF DM) trials (n=1,145) demonstrated that 71% of the participants exhibited elevated C-reactive protein levels (CRP ≥2 mg/L). Treatment with semaglutide led to consistent improvements in HF-related symptoms, exercise tolerance, and body weight reduction across the baseline CRP strata, along with a reduction in systemic inflammation, independent of the magnitude of weight loss [73].

Population prevalence projections were derived from age-specific incidence and survival modeling in large community cohorts, incorporating secular increases in obesity and diabetes; while absolute prevalence varies geographically, the metabolic drivers of HFpEF are consistent across sex and racial groups, with women and individuals with visceral adiposity exhibiting the highest lifetime risk [6,19,20]. Mechanistically, converging human biopsy, imaging, and biomarker data support an ‘outside-in’ paradigm in which systemic metabolic inflammation precedes myocardial dysfunction, distinguishing HFpEF from pressure-overload or purely cardiomyocyte-centric models [21,22,24]. Longitudinal evidence indicates a temporal cascade whereby visceral adiposity and insulin resistance initiate chronic low-grade inflammation, followed by endothelial activation, coronary microvascular rarefaction, impaired nitric oxide-Cyclic Guanosine Monophosphate (cGMP)-Protein Kinase G (PKG) signaling, and subsequent titin hypophosphorylation and diastolic stiffening [21,24,29,30]. Endothelial dysfunction is most reliably reflected by reduced coronary flow reserve, elevated inflammatory biomarkers (IL-6, CRP), and increased epicardial adipose tissue, each correlating with filling pressures and exercise intolerance [22,43,46,73].

Inflammatory biomarkers also offer details about disease mechanisms and potential stratification. In the MESA cohort (n=6,814), baseline IL-6, TNF-α, and CRP were associated with incident heart failure, with IL-6 and CRP specifically predicting HFpEF but not HFrEF or HFmrEF, suggesting a phenotype-specific association with systemic inflammation [74].

Clinical trials, including TOPCAT, demonstrated phenotypic heterogeneity by identifying specific HFpEF clusters: a younger demographic exhibiting preserved functional status and minimal LVH; an older group characterized by atrial fibrillation, concentric remodeling, and vascular stiffness; and a metabolically compromised cohort with obesity, diabetes, chronic kidney disease, concentric LVH, and elevated inflammatory biomarker levels. These phenotypes demonstrate differential risks for cardiovascular outcomes, highlighting the relevance of integrated clinical and biomarker-based stratification [75].

Proteomic and biochemical markers provided additional information. The TOPCAT study found that the urinary protein/creatinine ratio (UPCR) was linked to growth differentiation factor-15 (GDF-15), N-terminal pro-atrial natriuretic peptide (NT-proANP), adiponectin, fibroblast growth factor-23 (FGF-23), and soluble TNF receptors. FGF-23 was also found to be a separate predictor of HF hospitalization and death [76]. Serum albumin, an easily measured clinical parameter, has an inverse relationship with inflammation, liver fibrosis, arterial stiffness, diastolic dysfunction, pulmonary hypertension, and negative outcomes, retaining its prognostic significance even within the normal range [77]. Nutritional and hepatic markers, such as serum cholinesterase and plasma osmolality, also demonstrate prognostic relevance in acute HFpEF populations [78,79].

Emerging biomarkers, including circulating microRNAs and GDF-15, show promise in phenotyping and prognostication, but require validation in larger clinical cohorts. MicroRNA panels can differentiate between HFpEF and HFrEF, enhancing the diagnostic utility of conventional natriuretic peptides. GDF-15 levels, on the other hand, are linked to cardiac burden, renal dysfunction, inflammation, and nutritional status in elderly patients with multiple health problems, giving more prognostic information than traditional risk scores [80,81].

Collectively, these studies underscore that HFpEF cannot be treated as a uniform disease. Integrating metabolic, inflammatory, echocardiographic, and proteomic profiling allows for a deeper grasp of individual patient pathophysiology, moving beyond ejection fraction-based classification toward a precision medicine framework that accounts for comorbidities, systemic inflammation, and the myocardial metabolic status of the patient. Available human data indicate that SERCA2a impairment in HFpEF is modest compared with HFrEF, with preserved protein expression but functionally reduced calcium reuptake efficiency, supporting a role for impaired relaxation rather than overt systolic failure. Skeletal muscle dysfunction in HFpEF appears multifactorial, arising from systemic inflammation and physical deconditioning with contributory intrinsic mitochondrial abnormalities, rather than a single dominant mechanism. Consistent with this, exercise intolerance correlates more strongly with peripheral metabolic dysfunction and impaired oxygen utilization than with resting cardiac energetic deficits alone. 

How can we treat HFpEF by targeting metabolism?

HFpEF is now viewed as a cardiorenal-metabolic syndrome driven by visceral fat, insulin resistance, and chronic inflammation [24]. Correcting these disturbances is therefore central to therapy.

Foundational Therapy 

SGLT2 inhibitors (empagliflozin 10 mg or dapagliflozin 10 mg once daily) are first-line. EMPEROR-Preserved (n≈6, 000, EF≥40%) showed a 21% reduction in cardiovascular death or HF admission (HR 0.79; 95% CI 0.69-0.90) [10], and DELIVER gave similar results irrespective of diabetic status [23].

Weight-Centric Options

GLP-1 receptor agonists target adiposity-related dysfunction. In the STEP-HFpEF study (n=529), weekly semaglutide 2.4 mg improved the Kansas City Cardiomyopathy Questionnaire (KCCQ) score by approximately +8 points, increased the Six-Minute Walk Distance (6MWD) by +20 m, and reduced weight by 13% compared to placebo [82]; these benefits were also observed in diabetic patients in the STEP-HFpEF DM study [83]. Registry data (n≈14 000) suggest additive protection when GLP-1 RA is combined with SGLT2 inhibition [84].

Lifestyle Core 

A 400-450 kcal/day deficit plus ≥150 min/week of moderate aerobic exercise and two to three resistance sessions yields 7-10% weight loss and +2.5 mL/kg/min peak VO₂ [15]. According to phenotype, we use Mediterranean or Dietary Approaches to Stop Hypertension (DASH) patterns, high-protein hypocaloric plans, and very-low-calorie meal replacements while monitoring renal function and lipids [85]. Sodium (≈1.5-2 g/day) and fluid restriction, plus continuous positive airway pressure (CPAP) for obstructive sleep apnea, complete basic care.

Surgical/Emerging Options 

In a cohort of 12 patients with Class II-III obesity that was refractory to medical therapy, bariatric surgery (e.g., Roux-en-Y) resulted in approximately 30 kg of weight loss and reversed LVH within six months [86]. Ketone ester raised cardiac output 0.2 L/min and lowered pulmonary capillary wedge pressure (PCWP) by ≈5 mmHg during exercise in 24 patients [87], but metabolic modulators (trimetazidine, ranolazine) gave neutral or transient results [88,89]. Anti-IL-1 agents and colchicine lower biomarkers yet lack hard outcome benefit and remain investigational.

Practical Algorithm 

Euvolemic patients start an SGLT2 inhibitor, a loop diuretic, and evidence-based blood pressure (BP) control (MRA ± RAAS agent, target <130/80 mmHg). GLP-1 RA is added for BMI ≥30 kg/m² or metabolic syndrome/T2DM. Structured diet-exercise counseling aims for 7-10% weight loss over three to six months; bariatric surgery, ketone supplementation, trimetazidine, ranolazine, or anti-cytokine drugs are reserved for select cases or clinical trials [15,24,82-89].

Where do we go from here? (Future directions)

HFpEF is recognized as one of the most complex and heterogeneous entities in modern cardiology. Once viewed primarily as a disorder of diastolic relaxation, HFpEF is increasingly understood as a multisystem, metabolic-inflammatory condition involving the interplay of the heart, vasculature, adipose tissue, skeletal muscle, kidneys, and the immune system [24]. In clinical practice, patients present with clusters of comorbidities, including obesity, diabetes mellitus, hypertension, and chronic kidney disease, which converge on the mechanisms of systemic inflammation, oxidative stress, endothelial dysfunction, and impaired myocardial energetics [21,90]. EAT has emerged as a metabolically active endocrine organ that secretes pro-inflammatory cytokines, such as TNF-α and IL-6, directly promoting myocardial fibrosis and diastolic stiffening [91,92].

Emerging evidence has strengthened the adipokine hypothesis of HFpEF, particularly in the context of obesity- and metabolic-associated phenotypes. Dysfunctional adipose tissue in HFpEF is now recognized as an active endocrine organ that releases a maladaptive profile of adipokines, including increased leptin, resistin, and pro-inflammatory cytokines with relative adiponectin deficiency, thereby promoting systemic inflammation, coronary microvascular endothelial dysfunction, and myocardial fibrosis. This paradigm integrates well with the comorbidity-driven inflammatory model of HFpEF, wherein excess visceral and epicardial adipose tissue amplifies local and systemic inflammatory signaling, impairing nitric oxide-cGMP-PKG signaling and increasing cardiomyocyte stiffness. Importantly, adipokine-mediated paracrine effects from epicardial fat may directly influence adjacent myocardium, contributing to diastolic dysfunction independent of traditional hemodynamic load. Incorporation of the adipokine hypothesis thus provides a mechanistic link between obesity, metabolic dysregulation, and myocardial remodeling in HFpEF and underscores adipose tissue inflammation as a potential therapeutic target in this increasingly prevalent HFpEF phenotype [21,92]. 

In parallel, mitochondrial dysfunction within cardiomyocytes leads to impaired ATP production, increased production of ROS, contractile reserve failure, reinforcing fibrotic remodeling, and worsening ventricular compliance [52,93,94]. This shift in perspective redefines HFpEF as a systemic metabolic disease process rather than a localized cardiac “pumping problem,” mandating systemic diagnostic and therapeutic solutions. Therefore, the next phase of research must map these interconnected pathophysiological networks, translate them into early diagnostic tools, and design targeted therapeutic trials that reflect the biological diversity of HFpEF [24,90,95].

Reconceptualizing HFpEF as a systemic disorder emphasizes the necessity to examine the roles of adipose tissue dysfunction, chronic inflammation, vascular stiffness, insulin resistance, and mitochondrial impairment in disease progression [21,90]. Through its pro-inflammatory secretome, EAT perpetuates myocardial oxidative stress and fibrosis, whereas mitochondrial energetic failure exacerbates stiffness and impairs relaxation [91-94]. To capture this complexity, future studies should integrate multilayered biological data spanning genomics, proteomics, metabolomics, and advanced imaging. Early phenomapping studies have demonstrated that patients with HFpEF cluster into biologically distinct subgroups, each with different metabolic signatures and prognostic trajectories [95], offering a blueprint for precision medicine.

Table 3 outlines the key metabolic therapeutic trials in HFpEF.

**Table 2:** Key metabolic therapeutic trials in HFpEF EMPEROR-Preserved: EMPagliflozin outcomE tRial in Patients With chrOnic heaRt Failure With Preserved Ejection Fraction; DELIVER: Dapagliflozin Evaluation to Improve the Lives of Patients With Preserved Ejection Fraction Heart Failure; STEP-HFpEF: Semaglutide Treatment Effect in People with obesity and Heart Failure with Preserved Ejection Fraction; STEP-HFpEF DM: Semaglutide Treatment Effect in People With Obesity and HFpEF and Type 2 Diabetes Mellitus; DoPING-HFpEF: TrimetaziDine as a Performance-enhancING drug in Heart Failure with Preserved Ejection Fraction; RALI-DHF: RAnoLazIne for the Treatment of Diastolic Heart Failure; RCT: randomized controlled trial; HFpEF: heart failure with preserved ejection fraction; EF: ejection fraction; CV: cardiovascular; HR: hazard ratio; KCCQ: Kansas City Cardiomyopathy Questionnaire; 6MWD: 6-minute walk distance; VO₂: peak oxygen consumption; LV: left ventricle; T2DM: type 2 diabetes mellitus; PCWP: pulmonary capillary wedge pressure; CO: cardiac output; TID: three times daily; BID: twice daily; LVEDP: left ventricular end-diastolic pressure.

Study	Intervention	Population & design	Key results
Anker et al. (EMPEROR‑Preserved; 2021) [10]	Empagliflozin 10 mg once daily	RCT, HFpEF (EF ≥ 40%), n = 5,988	↓ CV death/HF hospitalization HR 0.79; HF hospitalization HR 0.71
Solomon et al. (DELIVER; 2022) [23]	Dapagliflozin 10 mg once daily	RCT, HFpEF (EF ≥ 40%), n = 6,263	↓ HF worsening/CV death HR 0.82
Kosiborod et al. (STEP‑HFpEF; 2023) [82]	Semaglutide 2.4 mg weekly	RCT, obese HFpEF (non‑diabetic), n = 529	↑ KCCQ +16.6 pts; weight –13.3%; 6MWD +21.5 m
Kosiborod et al. (STEP‑HFpEF-DM; 2024) [83]	Semaglutide 2.4 mg weekly	RCT, obese diabetic HFpEF, n = 616	KCCQ +13.7 pts; weight –9.8%
Kitzman et al. (2016) [15]	Diet + exercise	RCT, obese HFpEF, n = 100	VO₂ +2.5 mL/kg/min; weight –10%
Mikhalkova et al. (2018) [86]	Roux‑en‑Y gastric bypass	Cohort, obese HFpEF women, n = 12	↓ LV mass; ↑ diastolic function; NYHA class improved
Gopalasingham et al. (2024) [87]	Oral ketone ester (multiple daily doses; 2 weeks)	Crossover, HFpEF + T2DM, n = 24	CO +0.2 L/min; PCWP –5 mmHg
van de Bovenkamp et al. (DoPING‑HFpEF; 2023) [88]	Trimetazidine (20 mg TID)	Crossover, HFpEF, n = 25	No meaningful improvement
Maier et al. (RALI-DHF; 2013) [89]	Ranolazine (500 mg BID)	RCT; n=20, HFpEF	Transient ↓ LVEDP & PCWP; no lasting clinical benefit

A major unmet need remains the early diagnosis of HFpEF, as many patients present with irreversible myocardial and functional damage. Future research should prioritize the identification of metabolic biomarkers that serve as preclinical warning signs, including elevated epicardial fat burden, systemic insulin resistance, altered ketone metabolism, and pro-inflammatory cytokine signatures [96,97]. Novel diagnostic modalities, such as cardiac MRI for precise quantification of epicardial fat, plasma proteomics to detect circulating inflammatory mediators, and wearable biosensors capable of capturing exertional intolerance, hold promise as tools for earlier, noninvasive detection [97,98]. Longitudinal population-based cohort studies of individuals with high-risk phenotypes, such as obese or diabetic older adults with hypertension, are essential to validate these biomarkers and establish preventive and diagnostic strategies [32]. The goal is to build multimarker panels tailored for at-risk populations, thereby shifting HFpEF diagnosis toward a preventive and personalized model.

Table 4 highlights the metabolic biomarker candidates for early diagnosis of HFpEF.

**Table 3:** Metabolic biomarker candidates for the early diagnosis of HFpEF EAT: Epicardial Adipose Tissue; HFpEF: Heart Failure with Preserved Ejection Fraction; IL-6: Interleukin-6; CRP: C-Reactive Protein; MRI: Magnetic Resonance Imaging; CT: Computed Tomography; HOMA-IR: Homeostatic Model Assessment of Insulin Resistance.

Biomarker	Mechanistic link to HFpEF	Detection method
Epicardial adipose tissue (EAT) [91,92]	Pro-inflammatory, mechanical compression	Cardiac MRI, CT
IL-6/CRP [91,93]	Systemic inflammation and myocardial fibrosis	Plasma proteomics
Ketone bodies [87]	Impaired cardiac energetics and metabolism	Blood ketone panel
Insulin Resistance (HOMA-IR) [96,97]	Endothelial dysfunction, stiffening	Fasting insulin/glucose
Exertional intolerance [98]	Functional limitation from early diastolic dysfunction	Wearable sensors

Therapeutically, the evolving HFpEF model highlights inflammation, energy failure, and adipose dysfunction as critical domains for intervention. Current drugs, such as SGLT2 inhibitors and GLP-1 receptor agonists, although beneficial, were not originally developed with HFpEF as the primary target [10,82]. However, dedicated investigations are required to directly test biologically grounded therapies. Inflammation remains a key focus of IL-6 inhibitors, such as ziltivekimab, under evaluation in the Heart Failure Events Reduction with Remote Monitoring and eHealth Support (HERMES) trial, a large, randomized, double-blind study enrolling approximately 5,600 patients with HFpEF or HFmrEF and elevated hsCRP (≥2 mg/L) to determine whether IL-6 blockade can reduce hospitalizations, cardiovascular death, or urgent HF visits [99]. Trials of other anti-inflammatory agents, including colchicine and Nuclear Factor kappa-light-chain-enhancer of activated B cells (NF-κB) pathway modulators, have been registered across major platforms, such as the Clinical Trials Registry of India (CTRI) and ClinicalTrials.gov [100,101]. In parallel, therapies that restore myocardial energetics, including ketone ester supplementation, metabolic modulators such as trimetazidine, and mitochondria-targeted antioxidants, require rigorous evaluation in long-term outcome trials [87].

Finally, adipose-targeted strategies beyond systemic weight loss, such as pharmacological modulation of epicardial fat or minimally invasive interventions, including EAT ablation or localized anti-inflammatory delivery, represent an innovative frontier in addressing the mechanical and paracrine effects of dysfunctional fat on myocardial function [10]. The limitations of the current HFpEF trial methodology require further attention. Most existing studies have adopted a uniform design centered on LVEF thresholds and failed to capture the biological diversity of the syndrome. Future trials must employ stratified designs based on metabolic, inflammatory, or imaging-defined phenotypes to improve treatment response and decrease heterogeneity [90,95]. Phenotype-specific strategies encompass the administration of anti-inflammatory therapies to patients exhibiting elevated CRP or IL-6 levels, the use of GLP-1 receptor agonists in obese individuals with increased EAT, and the provision of ketone supplementation in insulin-resistant HFpEF subsets. Innovative trial structures, such as adaptive platform protocols, provide promising solutions. By enabling interim analyses, dynamic addition or removal of treatment arms, and biomarker-based stratification, these designs can improve trial efficiency and clinical relevance [102]. The success of Randomized Embedded Multifactorial Adaptive Platform for Community-acquired Pneumonia (REMAP-CAP) during the COVID-19 pandemic shows that the model can speed up the process of finding new treatments for complex syndromes [102]. Regulatory authorities, including the U.S. Food and Drug Administration (USFDA) and the European Medicines Agency (EMA) have acknowledged the utility of such frameworks, issuing guidance to promote their application in areas of high heterogeneity, such as oncology, rare diseases, and HFpEF [103]. The ultimate translational objective is to embed metabolic profiling into routine HFpEF care, analogous to the current use of cholesterol or HbA1c in cardiovascular and diabetes management. Risk stratification tools that integrate clinical parameters with imaging-derived and biochemical biomarkers could enable both early diagnosis and personalized treatment selection [97]. Digital health is poised to catalyze this transformation; artificial intelligence-driven phenotyping now applies machine learning to large-scale omics and imaging datasets, uncovering subtle HFpEF subgroups with predictive value for prognosis and therapy [104]. Simultaneously, wearable biosensors, such as ECG patches, smartwatches, and ambulatory hemodynamic monitors, allow real-time tracking of physical activity, heart rate, and fluid status in everyday settings [105,106]. These technologies may enable dynamic risk profiling, early decompensation detection, and individualized therapy titration. To support such advances, clinical training programs must update their curricula to reflect the metabolic and digital dimensions of HFpEF. Multidisciplinary collaboration among cardiologists, endocrinologists, nephrologists, and primary care providers is essential, particularly within specialized HFpEF clinics serving patients with overlapping comorbidities of obesity, diabetes, and heart failure [107,108]. 

Conceptualizing HFpEF as an ‘outside-in’ metabolic-inflammatory syndrome superimposed on ‘inside-out’ mitochondrial energetic failure redirects therapeutic focus from isolated afterload reduction toward metabolic modulation, including SGLT2-mediated ketone utilization, GLP-1 receptor agonist-induced weight reduction, and exercise-driven mitochondrial biogenesis, providing a mechanistic framework for phenotype-targeted trials [24,30,82].

## Conclusions

This review synthesizes emerging evidence positioning HFpEF as a heterogeneous systemic condition driven by metabolic inflammation, adipose tissue dysfunction, endothelial impairment, and mitochondrial energy failure, shifting the paradigm away from its historical classification as an isolated cardiac filling disorder. By integrating findings from mechanistic studies, large-scale clinical trials, and epidemiological cohorts, it becomes clear that HFpEF is best conceptualized as a cardio-metabolic syndrome in which comorbidities such as obesity, diabetes, and hypertension act as central drivers of disease progression. Despite advances with therapies such as SGLT2 inhibitors and GLP-1 receptor agonists, significant limitations remain, including heterogeneity in study designs, underrepresentation of diverse populations, and reliance on observational data for key mechanistic insights. Addressing these gaps will require large, longitudinal cohorts, biomarker-guided stratification, and adaptive clinical trial designs that specifically target metabolic, inflammatory, and energetic dysfunction. Future research must also embrace precision medicine tools, including omics-based profiling, advanced imaging, and digital health technologies, to improve early detection and tailor interventions. Ultimately, redefining HFpEF as a systemic metabolic heart disease offers the opportunity to move beyond symptom management toward interventions that modify its underlying biology, paving the way for more effective and individualized care.
